# Bead-Like Pt/C-Ionomer Porous Nanofibrous Networks Toward Advanced Electrochemical Reaction Management for Direct Methanol Fuel Cells

**DOI:** 10.3390/membranes15120362

**Published:** 2025-11-29

**Authors:** Ruili Sun, Dongming Zhu, Nan Wu, Yi Li, Ting Chen, Shaorong Wang

**Affiliations:** 1 School of Chemical Engineering & Technology, China University of Mining and Technology, Xuzhou 221116, China; 15978473579@163.com (D.Z.); pilgrimxiaoe@foxmail.com (Y.L.); 2 Suzhou Institute for Advanced Research, University of Science and Technology of China, Suzhou 215123, China; keii@mail.ustc.edu.cn

**Keywords:** direct methanol fuel cells, electrode architecture, nanofibrous electrode

## Abstract

Efficient management for electrochemical reactions within Pt/C electrodes, specifically the oxygen reduction reaction (ORR) and methanol oxidation reactions (MOR), is critical to the performance and long-life stability of direct methanol fuel cells (DMFCs). Optimizing the hierarchical macro/mesoscale structures of Pt/C electrodes plays a decisive role in regulating the mass transport pathways and electrochemical reactions. In this work, bead-like Pt/C-ionomer hybrid porous nanofibrous networks are constructed via electrospinning. Ascribing to the hierarchical architecture consisting of continuous nanofibers and bead-like Pt/C-ionomer fibrous networks, the hybrid porous nanofibrous electrode exhibits a 55% increase in maximum mass power density in comparison to the conventional Pt/C electrode. Such enhancement is attributed to excellent ORR activity enabled by efficient triple-phase reaction regions, coupled with superior MOR tolerance resulting from restricted methanol transport from the hybrid porous nanofibrous electrode to triple-phase reaction regions.

## 1. Introduction

Direct Methanol Fuel Cells (DMFCs) have been considered as one of the promising hydrogen-energy utilization technologies for addressing the challenge of fossil fuel consumption, with the advantage of high energy-conversion efficiency, environmental friendliness, and quick start-up [1,2,3,4]. However, the cost issues of the massive utilization of the scarce Pt-based electrocatalyst still hinder the commercial availability of DMFCs [5]. Although many non-precious metal electrocatalysts have been constructed, Pt-based electrocatalysts are still regarded as the best materials for the oxygen reduction reaction (ORR) and methanol oxidation reaction (MOR) of DMFCs [6,7,8]. Nevertheless, even if assembled with high-activity Pt-based electrocatalysts under high Pt loadings, the output performance of DMFCs has not satisfied the DOE department target (>250 mW cm^−2^), which could be due to the complex electrochemical reactions occurring within Pt/C electrodes, the toxicity of the CO intermediate product, and the methanol or oxygen transport resistance [9,10,11,12]. Especially, the coexistence of ORR and MOR on the cathode of DMFCs could bring out a hybrid potential, which severely depresses the cathode potential and consumes the oxygen for MOR. Simultaneously, the CO intermediate species generated from MOR could have a strong adsorption for the Pt-based electrocatalyst surface, directly affecting the electrochemical surface area (ECSA) of the Pt-based electrocatalysts. Such events drastically compromise the output performance and long-term durability of DMFCs. Hence, optimizing electrochemical reactions for the cathode of DMFCs could become an effective strategy for reducing Pt-based electrocatalyst loadings without sacrificing the output performance and long-term durability of DMFCs.

As is well known, conventional Pt/C electrodes are composed of Pt-based electrocatalysts, ionomer materials, and void regions, which collectively form triple-phase boundary regions and mass transport pathways [13,14]. However, the random and tortuous pore networks significantly hinder the mass transport processes, resulting in considerable concentration polarization at the high current density in DMFCs [15,16]. These conventional structures also fail to effectively block methanol permeation from the anode through the Nafion membrane, leading to undesired MOR and exacerbating the mixed potential issue at the cathode [17,18]. Moreover, the uneven and ineffective triple-phase boundary regions facilitate both ORR and MOR, thereby reducing the output potential and performance of Pt/C electrodes [19]. Therefore, optimizing the hierarchical macro/mesoscale structure of Pt/C electrodes represents a promising strategy to enhance electrochemical reactions by facilitating the oxygen transport properties and suppressing the methanol transport process without increasing the loading of Pt-based electrocatalysts.

Ordered structured electrodes have emerged as a promising approach for constructing Pt/C electrodes with hierarchical macro/mesoscale architectures [20]. Among various fabrication techniques, electrospun nanofiber electrodes have gained attention due to the advantage of facile preparation, tunable hierarchical structures, and well-defined pathways for mass, electron, and proton transport [21,22,23]. Previous studies have evidenced outstanding mass transport properties and single-cell performance of nanofiber electrodes in comparison to conventional Pt/C electrodes, which is attributed to the ordered conducive pathways, effective mass transport channels, and high electrocatalyst utilization in the nanofiber electrodes [24,25]. For example, Pintauro et al. have fabricated a Pt/C nanofiber electrode by electrospinning an ink consisting of Pt/C electrocatalysts, ionomer materials, and polyacrylic acid (PAA) materials [26]. The single cell assembled with this nanofiber electrode achieves a peak power density of approximately 524 mW cm^−2^ with a 1.6-fold enhancement over conventional Pt/C electrodes, attributed to a larger ECSA and reduced oxygen-transport resistance. Furthermore, Shao et al. have developed a free-standing and ionomer-free fiber network electrode by electrospinning and physical vapor deposition [27]. This electrode delivers a peak power density of 0.936 W cm^−2^, benefiting from enhanced reactant transport properties and water management enabled by the hierarchical structures. Apparently, such hierarchical structures of Pt/C electrodes have been applied as the cathode of proton-exchange membrane fuel cells (PEMFCs). These structures are shown to facilitate rapid O_2_ diffusion to the active sites, which contributes to the superior output performance of PEMFCs [28,29,30]. Collectively, these studies provide valuable insights into the broader application of electrospinning for electrode fabrication and demonstrate the benefits of nanofiber electrodes in enhancing oxygen transport within the cathode of PEMFCs. Given the similarity between the cathodes of PEMFCs and DMFCs, it has been proposed that hierarchical structures of Pt/C electrodes could be suitably employed in the cathode of DMFCs. However, their application as cathodes in DMFCs remains largely unexplored. This could be attributed to the fact that the effect of the hierarchical macro/mesoscale structures on the methanol transport processes and MOR is still not unclear, while many researchers have proposed that a continuous, ordered scaffold could effectively suppress methanol permeation. Hence, a systematic investigation into designing the hierarchical macro/mesoscale structures of Pt/C electrodes and exploring the effect of these structures on the complex electrochemical reactions and mass transport processes in DMFCs is urgently needed.

In this work, we report a hierarchically ordered electrode composed of bead-like Pt/C-ionomer hybrid porous nanofiber networks via electrospinning and further explore the application of the hierarchically ordered electrode in the cathode of DMFCs. The morphology and composition of the electrode are investigated by field-emission scanning electron microscopy, field-emission transmission electron microscopy, thermogravimetric analysis, and X-ray photoelectron spectroscopy. The electrochemical behaviors toward ORR and MOR for the hierarchically ordered electrode are systematically evaluated in DMFCs. Furthermore, the correlation between the hierarchical macro/mesoscale structures within the novel Pt/C electrode and its electrocatalytic activity for ORR and MOR in DMFCs is elucidated. These insights are expected to facilitate the development of DMFCs toward high performance and long-life stability.

## 2. Materials and Methods

### 2.1. Fabrication of the Pt/C–PAA–Nafion Electrode and Conventional Pt/C Electrode

Pt/C–PAA–Nafion electrodes are fabricated via electrospinning. In detail, 60 wt.% Pt/C (50 mg, Johnson Matthey, Royston, UK) is added into 0.5 mL of deionized water and then magnetically stirred for about 1 h. Polyacrylic acid (25 mg, PAA, Mw: 420,000 g mol^−1^, Aladdin, Shanghai, China) is added to a Nafion ionomer solution (1 mL, 5 wt.%, Dupont, Wilmington, DE, USA) and then magnetically stirred for about 1 h. The above two solutions are mixed and then magnetically stirred for about 12 h. This homogeneous solution is placed in a 2 mL syringe. A voltage of 10~12 kV with a feeding speed (0.1 mL h^−1^) is applied between a stainless needle and a collector. This sample on the collector is under a vacuum-drying process at 40 °C for about 12 h, and then a Pt/C–PAA–Nafion electrode is obtained. In the fabrication process, PAA materials play the role of electrospinning polymer materials for ensuring the form of the nanofiber in this electrode, and the Pt/C and ionomer materials are the key components for constructing the triple-phase interfacial areas. For comparison, the baseline electrode, namely the conventional Pt/C electrode, is fabricated using the spraying method. It is worth noting that a variety of techniques, such as bar coating, screen printing, and roll-to-roll processing, could be employed to prepare the conventional Pt/C electrode. However, considering the complexity of fabricating processes and their impact on the performance of DMFCs, spraying stands out as a straightforward and suitable method for preparing small-area electrodes in a student laboratory setting. Hence, the conventional Pt/C electrode is fabricated via spraying the ink, consisting of commercial 60 wt.% Pt/C (Johnson Matthey, Royston, UK), Nafion ionomer solutions, deionized water, and ethanol, onto the SGL29BC GDL, with a Pt loading of 0.788 mg cm^−2^.

### 2.2. Physical Characterizations

Field-emission scanning electron microscopy (FSEM, M0AIA3 LMH, TESCAN, Brno, Czech Republic), field-emission transmission electron microscopy (FTEM, Tecnao G2 F20, Thermo Fisher Scientific, Waltham, MA, USA), thermogravimetric (TG, Netzsch Co. Selb, Bavaria, Germany), and X-ray photoelectron spectroscopy (XPS, ESCALAB 250Xi, Thermo Fisher Scientific, Waltham, MA, USA) are applied to obtain the morphology, structures, and composition of the Pt/C–PAA–Nafion electrode. Contact-angle measurement is conducted to achieve the methanol behavior of the Pt/C–PAA–Nafion electrode and the traditional Pt/C electrode.

### 2.3. Conductivity Measurements

The effective electrode conductivity and effective proton conductivity of the Pt/C–PAA–Nafion electrode and conventional Pt/C electrode are evaluated according to the method described in our previous work [31]. This method is different from the blocked-electrode EIS method, which can directly separate the membrane and catalyst-layer ionic conductivities. In the electrode conductivity equipment, these electrodes with different thicknesses are placed in two pieces of flexible graphite as the electron-conducting layers, and there exist two sealing layers to ensure the electron transport in the electron-conducting layers. Electrochemical impedance spectroscopy (EIS) is employed in this measuring equipment under an amplitude of 5 or 10 mV. Furthermore, based on the ohmic resistance, the thickness, and the area of different electrodes, the electrode conductivity is obtained via the following equations:

*R*_electrode_ = *R*_2_ − *R*_1_

$$G_{e}=\frac{L}{R_{\mathrm{electrode}}\times S}$$

where *R*_1_ (mΩ) is the resistance of the measurement equipment for thinner samples, *R*_2_ (mΩ) is the resistance of the measurement equipment for samples with thicker samples, *L* (m) and *S* (m^2^) are the thickness and the area of testing samples, and *G*_e_ (mS m^−1^) is the electrode conductivity of testing samples.

For proton conductivity, the samples are placed in two pieces of Nafion^®^ 212 membrane as the proton-conducting layers, and there exist Pt/C electrodes for electrochemical reactions and two sealing layers for assuring the protons migrate in the proton-conducting layers. The measuring equipment is supplied with 50 mL min^−1^ humidified hydrogen at the cathode and anode sides, and EIS is employed in this measuring equipment under an amplitude of 5 or 10 mV. The proton conductivity is calculated via the following equation:

*R*_proton_ = *R*_4_ − *R*_3_

$$G_{p}=\frac{L}{R_{\mathrm{proton}}\times S}$$

where *R*_3_ (mΩ) is the resistance of the measurement equipment for thinner samples, *R*_4_ (mΩ) is the resistance of the measurement equipment for samples with thicker samples, *L* (m) and *S* (m^2^) are the thickness and the area of testing samples, and *G*_p_ (mS m^−1^) is the proton conductivity of testing samples.

### 2.4. Fabrication of MEAs

The Pt/C–PAA–Nafion electrode or conventional Pt/C electrode is applied as the cathode of MEAs. An anode is prepared by spraying the ink, consisting of commercial 75 wt.% PtRu/C (TKK, Tokyo, Japan), Nafion ionomer solutions, deionized water, and ethanol, onto the SGL29BC GDL, with a PtRu loading of 3.88 mg cm^−2^. The MEAs with 1 × 1 cm^2^ are assembled via pressing the Nafion^®^212 membranes sandwiched between the cathode and the anode at 135 °C with 4.6 kPa for 2 min, which are denoted as MEA Pt/C-PAA–Nafion and MEA-Pt/C.

### 2.5. DMFC Single-Cell Measurement

The performance of MEAs is evaluated via a homemade fuel cell system with an electrochemical station. During the CV measurement of the cathode (CCV), the cathode of MEAs is considered as the working electrode supplied with 1.0 mL min^−1^ deionized water, and the anode is fed with hydrogen at a flow rate of 10 mL min^−1^, respectively, in which the potential ranges from 0 V to 1.2 V vs. DHE at a scan rate of 20 mV s^−1^. During the CV test of the anode (ACV), the anode of MEAs is considered as the working electrode supplied with 1.0 mL min^−1^ deionized water, and the cathode is fed with hydrogen with a flow rate of 10 mL min^−1^, respectively, in which the potential ranges from 0 V to 0.7 V vs. DHE at a scan rate of 20 mV s^−1^. Methanol-crossover curves of MEAs are obtained via the linear sweep voltammetry (LSV) measurement, in which the cathode is regarded as the working electrode supplied with nitrogen at a flow rate of 30 mL min^−1^ and the anode is fed with 0.5 M methanol solution with the potential linearly ranging from 0 V to 0.6 V at a scan rate of 1 mV s^−1^. IV and EIS tests are carried out on MEAs under the cathode fed with air at a flow rate of 80 mL min^−1^ and the anode applied with 0.5 M methanol solution at a flow rate of 1.0 mL min^−1^. The alternating current (AC) potential frequency range is 1000 kHz to 0.1 Hz with a 10% amplitude at a current density of 100 mA cm^−2^. The ohmic-resistance (IR)-corrected polarization curves are obtained based on the IV curves and EIS results. Specifically, the corrected potential is calculated by adding the ohmic polarization loss, derived from the current multiplied by the ohmic resistance of the single cell obtained from the EIS results, to the measured potential of the IV curves. All electrochemical measurements could be conducted in more than three independent replicate tests, and the stable data could be displayed in the figures of this manuscript.

## 3. Results

### 3.1. Fabrication of the Pt/C–PAA–Nafion Electrode

**Figure 1:**
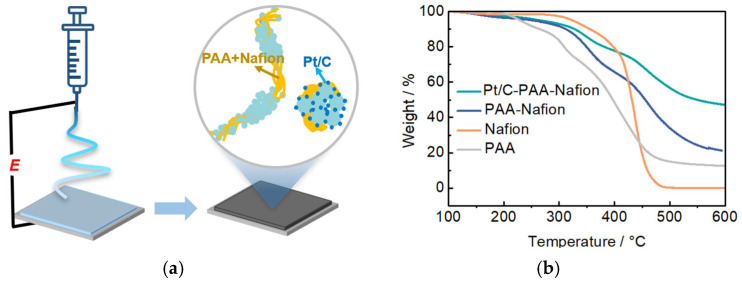
Schematics of the fabricating process for the Pt/C–PAA–Nafion electrode (**a**); TG results for the Pt/C–PAA–Nafion electrode (**b**).

Figure 1a illustrates the fabrication process of the Pt/C–PAA–Nafion electrode. The electrode is constructed via electrospinning an ink consisting of 60 wt.% Pt/C electrocatalysts, Nafion ionomer materials, and PAA materials, followed by vacuum drying at 40 °C for about 12 h. The thermal behavior and composition of the Pt/C–PAA–Nafion electrode are analyzed by TG measurements, as demonstrated in Figure 1b. The TG curves of the Pt/C–PAA–Nafion electrode, the PAA–Nafion sample, and the PAA materials indicate that the weight-loss events observed above 200 °C for Pt/C-PAA–Nafion are associated with the decomposition of PAA materials, while those above 300 °C correspond to the degradation of Nafion materials. These results confirm the presence of PAA and Nafion materials in the Pt/C–PAA–Nafion electrode and the PAA–Nafion sample. Furthermore, the TG curve of both the Pt/C–PAA–Nafion electrode, PAA materials, and Nafion materials demonstrates four distinct weight loss stages: removal of the adsorbed water below 150–200 °C; decomposition of PAA materials and Nafion materials around 200 °C and 300 °C; and the pyrolysis stage for Pt/C electrocatalysts occurring between 400 and 450 °C. Based on the residual mass difference between Pt/C-PAA–Nafion and PAA–Nafion samples, the Pt/C electrocatalyst content is obtained to be 49.6%, corresponding to a Pt loading of 0.68 mg cm^−2^ for the Pt/C–PAA–Nafion electrode, which is slightly lower than that of the conventional Pt/C electrode (0.788 mg cm^−2^). This indicates the comparable Pt loadings in the Pt/C-PAA–Nafion and conventional Pt/C electrodes, and the Pt loading might not be related to the electrochemical behaviors of the corresponding DMFCs.

### 3.2. The Morphology of the Pt/C–PAA–Nafion Electrode

**Figure 2:**
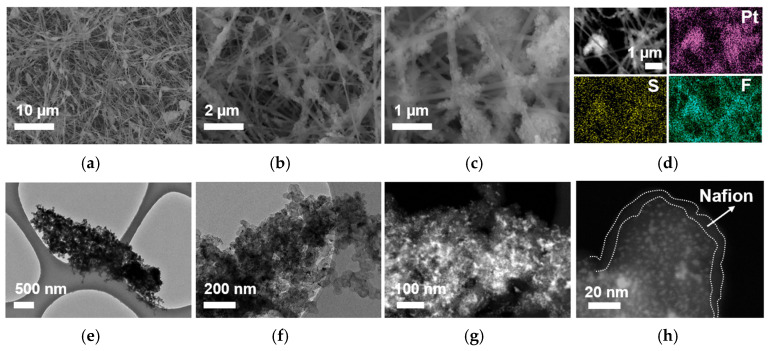
FSEM images (**a**–**c**), EDS images (**d**), and FTEM (**e**–**h**) images for the Pt/C–PAA–Nafion electrode.

The morphology of the Pt/C–PAA–Nafion electrode is characterized by FSEM with EDS mapping and FTEM, as displayed in Figure 2a–h. From the FSEM images (Figure 2a–c), the Pt/C–PAA–Nafion electrode exhibits the entangled porous nanofiber networks, in which continuous smooth nanofibers and bead-like fibrous nanofibers are observed. EDS mapping images (Figure 2d) confirm the uniform distribution of platinum, sulfur, and fluorine elements, indicating uniform incorporation of Pt/C electrocatalysts and Nafion ionomer materials, thus forming the bead-like Pt/C–ionomer nanofibers. Furthermore, from the FTEM images (Figure 2e–h), these bead-like structures exhibit the porous aggregates composed of Pt/C electrocatalysts coated with a thin film of Nafion ionomer materials. Based on the particle size analyzer, the average size of the porous aggregates could be calculated to be about 900 nm, in which the Pt particles exhibit an average diameter of 3~4 nm and the Nafion ionomer film thickness ranges from 2 nm to 15 nm. Such microstructure establishes efficient Nafion/Pt triple-phase reaction regions conducive to ORR and MOR. Additionally, the uniform distribution of sulfur and fluorine elements, along with the presence of PAA materials as electrospinning polymer, suggests that both PAA materials and Nafion ionomer materials constitute the continuous smooth nanofibers. These nanofibers are expected to serve as proton-conducting pathways, facilitating proton transport from the Nafion membrane to electrochemical reaction sites within the Pt/C–PAA–Nafion electrode. The above results have demonstrated that the Pt/C–PAA–Nafion electrode with the hierarchical macro/mesoscale structures has been fabricated, and thus the significant influence on electrochemical reactions and mass transport properties could be further explored.

### 3.3. The Structure of the Pt/C–PAA–Nafion Electrode

**Figure 3:**
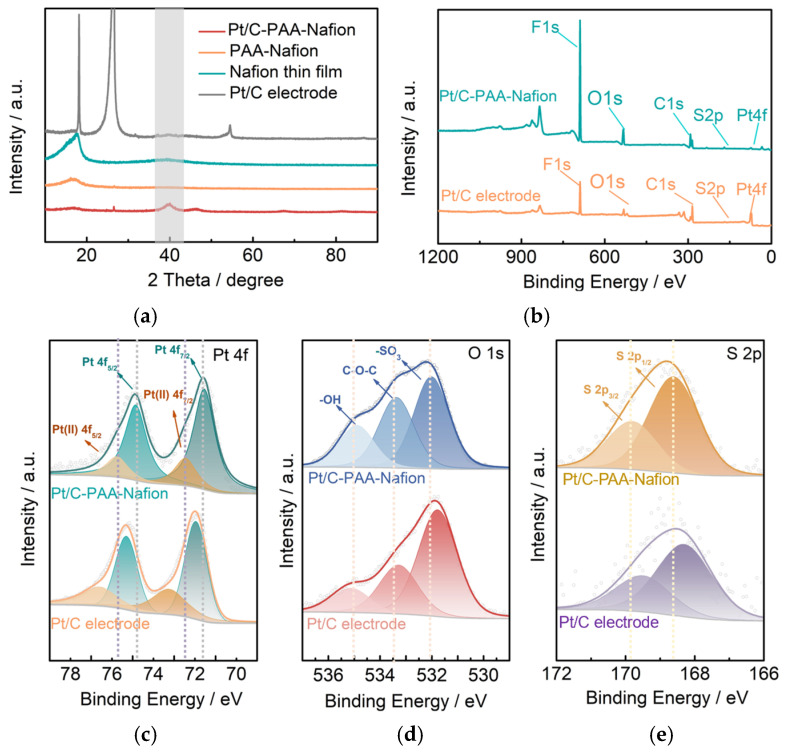
XRD pattern (**a**) and XPS spectra (**b**–**e**) of the Pt/C–PAA–Nafion electrode: XPS spectra of Pt 4f orbital (**c**), O 1s orbital (**d**), and S 2p orbital (**e**). The dashed lines (Figure 3c) represent the bonding energy sites of four electron states of Pt 4f spectra in the Pt/C–PAA–Nafion electrode. The dashed lines (Figure 3d) exhibit the bonding energy sites of –OH, C–O–C, and –SO_3_ groups in the Pt/C–PAA–Nafion electrode. The grey area is related to the (111) planes of Pt (PDF#04-0802). The dashed lines (Figure 3e) are the site of the bonding energy of the two electron states for the S 2p spectra in the Pt/C-PAA–Nafion electrode.

To analyze the crystalline phases of the Pt/C–PAA–Nafion electrode, the PAA–Nafion electrode, the Nafion thin film, and the conventional Pt/C electrode, XRD characterization is carried out. As illustrated in Figure 3a, the diffraction peak at 39.8° in the patterns of the Pt/C–PAA–Nafion electrode and the conventional Pt/C electrode is attributed to the (111) planes of Pt (PDF#04-0802), revealing the similar metallic fcc-structured Pt in these electrodes. Based on these diffraction peaks, the average diameter of Pt particles is calculated to be 3.78 nm and 3.69 nm for the Pt/C–PAA–Nafion electrode and the conventional Pt/C electrode, consistent with the FTEM results. This suggests the unchanged Pt crystalline phase in the Pt/C–PAA–Nafion electrode and the conventional Pt/C electrode, even if Pt/C electrocatalysts are processed using electrospinning technology. Further, the successful introduction of Nafion-ionomer materials is observed due to the diffraction peak at 17.7° in the patterns of the Pt/C–PAA–Nafion electrode, the PAA–Nafion electrode, and the Nafion thin film. Such an event suggests the preservation of Nafion ionomer materials in the Pt/C–PAA–Nafion electrode. Hence, the crystalline phases and structure of the Pt/C–PAA–Nafion electrode are not influenced by the electrospinning operation, and this might not affect the electrochemical behaviors of the corresponding DMFCs.

To explore the effect of the electrode fabrication processes, the surface chemical states and electronic interactions in the Pt/C–PAA–Nafion electrode and the conventional Pt/C electrode are analyzed via XPS, as shown in Figure 3. The full spectra presented in Figure 3b reveal the presence of F, O, C, S, and Pt elements in both Pt/C electrodes, confirming the similar composition. The Pt 4f spectra shown in Figure 3c exhibit the deconvoluted Pt 4f spectra, which are fitted with four electron states of Pt (II) 4f_5/2_, Pt 4f_5/2_, Pt (II) 4f_7/2_, and Pt 4f_7/2_. As summarized in Table 1, the Pt 4f peaks in the Pt/C–PAA–Nafion electrode exhibit negative-binding-energy shifts of 0.9 eV, 0.45 eV, 0.85 eV, and 0.40 eV, respectively, relative to those in the conventional Pt/C electrode. These shifts suggest stronger electronic interactions between Nafion ionomer materials or PAA materials and Pt/C electrocatalysts in the Pt/C–PAA–Nafion electrode. The O 1s spectra (Figure 3d) are deconvoluted into three components attributed to the signals of -OH, C-O-C, and -SO_3_ groups from Nafion-ionomer materials and PAA materials, and the bonding energy is displayed in Table 1. Compared to the conventional Pt/C electrode, the Pt/C–PAA–Nafion electrode shows a 0.25 eV downshift in the -OH peak, along with upshifts of 0.05 eV and 0.20 eV for the C-O-C and -SO_3_ peaks, respectively (Table 1). Combined with the Pt 4F spectra results, these shifts indicate possible electron transfer between the functional groups and Pt/C electrocatalyst via the connection of the Pt-O-S bond in the Pt/C–PAA–Nafion electrode. This statement could be further evidenced via the upshift bonding energy of S 2p_3/2_ and S 2p_1/2_ peaks (Figure 3e, Table 1). Hence, the electronic configuration of Pt/C electrocatalysts within the Pt/C–PAA–Nafion electrode is significantly adjusted by electrospinning, and these electronic alterations are expected to influence the electrochemical behavior of the Pt/C–PAA–Nafion electrode, particularly given the unique hierarchical macro/mesoscale architecture.

### 3.4. Single-Cell Performance of the Pt/C–PAA–Nafion Electrode

**Figure 4:**
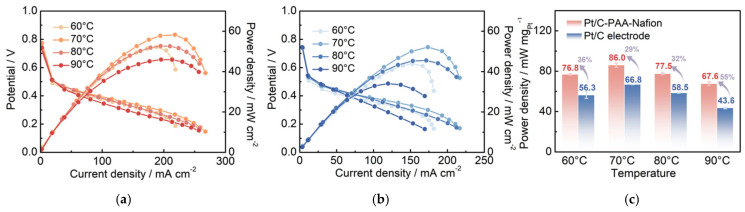
The polarization curves of the Pt/C–PAA–Nafion electrode (**a**) and the conventional Pt/C electrode (**b**); the peak power density of the Pt/C–PAA–Nafion electrode and the conventional Pt/C electrode (**c**). Testing conditions: the cathode is fed with air at a flow rate of 80 mL min^−1^, and the anode is fed with 0.5 M CH_3_OH at a flow rate of 1.0 mL min^−1^.

To further investigate the electrochemical performance of different Pt/C electrodes in DMFCs, the Pt/C–PAA–Nafion electrode and the conventional Pt/C electrode are assembled as cathodes and evaluated by the homemade measurement equipment. The corresponding IV curves are shown in Figure 4a,b. The peak power density of the single cell assembled with the Pt/C–PAA–Nafion electrode is 52.2, 58.3, 52.7, and 45.9 mW cm^−2^, respectively, while that of the conventional Pt/C electrode is 43.9, 52.1, 45.5, and 34.0 mW cm^−2^, respectively, across the testing temperatures. Notably, the single-cell output performance of both Pt/C samples exhibits a fluctuating trend as the operating temperature increases from 60 °C to 90 °C. The highest peak-power density is found at 70 °C, where the Pt/C–PAA–Nafion electrode achieves a 35% enhancement over the conventional Pt/C electrode. Considering the variation in Pt loadings between the two electrodes, the mass-specific power density of the cathode is calculated, as displayed in Figure 4c. The results clearly demonstrate that the Pt/C–PAA–Nafion electrode delivers a significantly higher mass-specific power density across all testing temperatures compared to the conventional Pt/C electrode. The maximum value is 86.0 mW mg_Pt_^−1^ at 70 °C for the Pt/C–PAA–Nafion electrode, with the greatest enhancement of 55% obtained at 90 °C. Given that the anode, the electrolyte membrane, and the testing conditions remain identical, the superior electrochemical performance in DMFCs should be related to the unique structure and electrochemical properties of the Pt/C–PAA–Nafion electrode.

## 4. Discussion

### 4.1. Analysis of the Single Cell Performance of DMFCs with the Pt/C–PAA–Nafion Electrode

**Figure 5:**
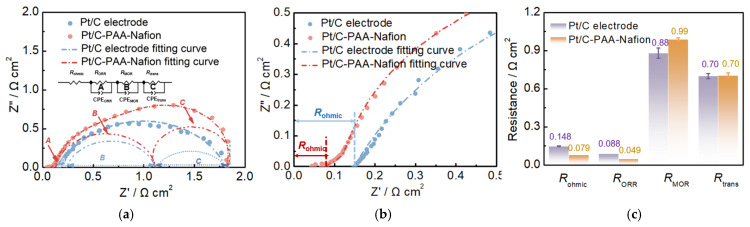
EIS results (**a**,**b**) and related fitting parameters (**c**) of DMFCs. Testing conditions: the cathode is fed with air at a flow rate of 80 mL min^−1^, the anode is fed with 0.5 M CH_3_OH at a flow rate of 1.0 mL min^−1^, and the current density is 100 mA cm^−2^.

**Figure 6:**
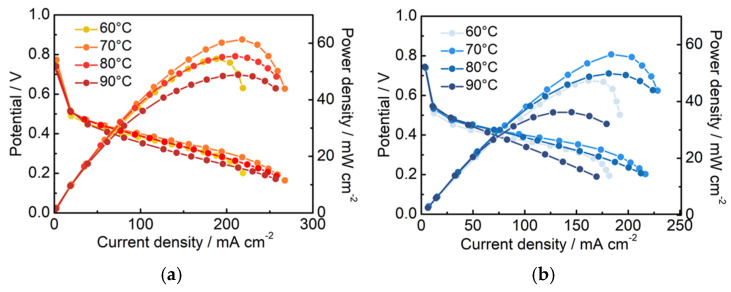
IR-corrected polarization curves for the Pt/C–PAA–Nafion electrode (**a**) and the conventional Pt/C electrode (**b**). Testing conditions: the cathode is fed with air at a flow rate of 80 mL min^−1^, and the anode is fed with 0.5 M CH_3_OH at a flow rate of 1.0 mL min^−1^.

**Figure 7:**
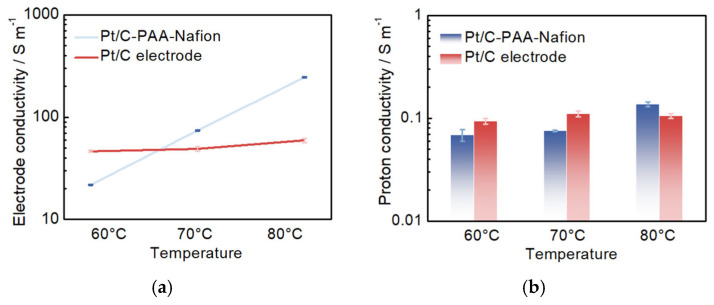
The electrode conductivity (**a**) and proton conductivity (**b**) for the Pt/C–PAA–Nafion electrode and the conventional Pt/C electrode.

**Figure 8:**
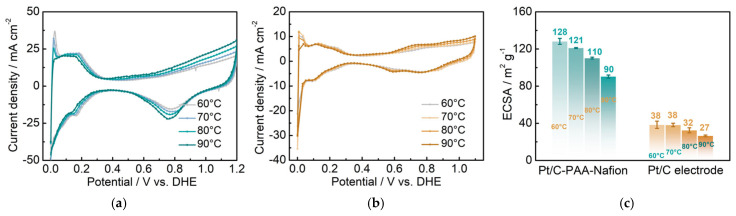
CV curves for DMFCs assembled with the Pt/C–PAA–Nafion electrode (**a**) and the conventional Pt/C electrode (**b**); ECSA for different electrodes (**c**). Testing conditions: the anode is fed with hydrogen at a flow rate of 10 mL min^−1^, and the cathode is fed with deionized water at a flow rate of 1.0 mL min^−1^.

**Figure 9:**
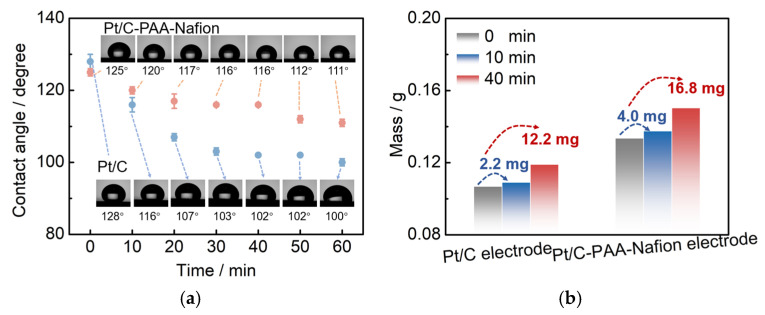
Contact angle of the Pt/C–PAA–Nafion electrode and the conventional Pt/C electrode (**a**); the mass of the methanol solution within the Pt/C–PAA–Nafion electrode and the conventional Pt/C electrode (**b**).

**Figure 10:**
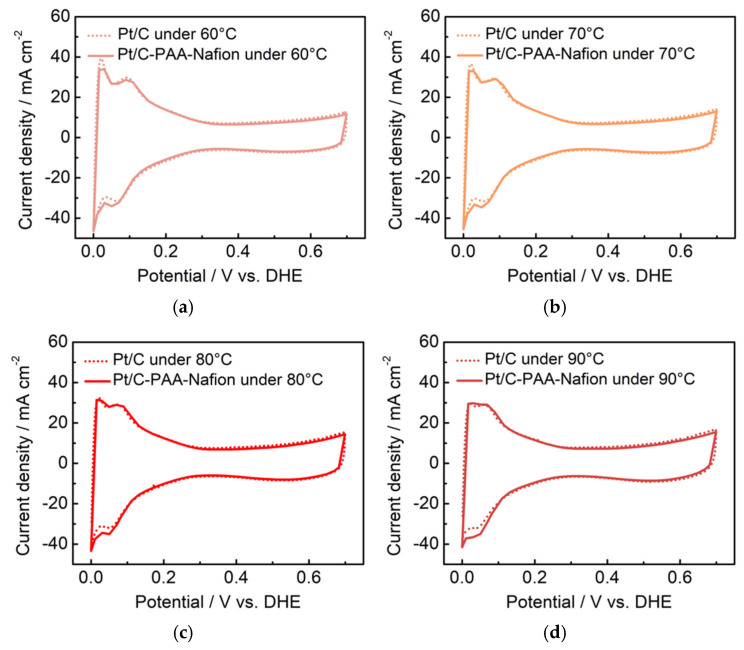
CV curves for the anode of DMFCs with the Pt/C–PAA–Nafion electrode and the conventional Pt/C electrode. The testing temperatures are 60 °C (**a**), 70 °C (**b**), 80 °C (**c**), and 90 °C (**d**). Testing conditions: the cathode is fed with hydrogen at a flow rate of 80 mL min^−1^, and the anode is fed with deionized water at a flow rate of 1.0 mL min^−1^.

**Figure 11:**
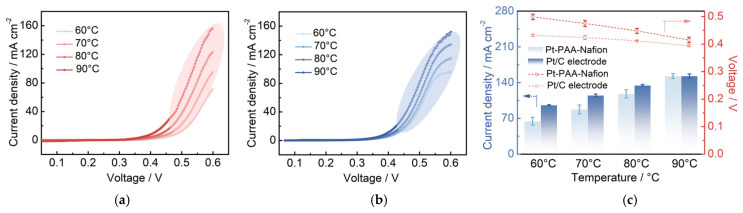
LSV profiles for the anode of DMFCs with the Pt/C–PAA–Nafion electrode (**a**) and the conventional Pt/C electrode (**b**). MOR parameters for the Pt/C–PAA–Nafion electrode and the conventional Pt/C electrode (**c**). Testing conditions: the cathode is fed with nitrogen at a flow rate of 30 mL min^−1^, and the anode is fed with 0.5 M methanol solution at a flow rate of 1.0 mL min^−1^.

Detailed analysis of the discrepancy in the output performance of DMFCs assembled with the Pt/C–PAA–Nafion electrode and the conventional Pt/C electrode is carried out via electrochemical characterizations, and the results are shown in Figure 5, Figure 6, Figure 7, Figure 8, Figure 9, Figure 10 and Figure 11. The EIS results of different DMFCs are presented in Figure 5a, in which the equivalent circuit diagrams are used to obtain the different resistances of electrochemical reactions and mass transport processes. Apparently, the total resistance of DMFCs has been divided into four components, which are the ohmic resistance of DMFCs (*R*_ohmic_), the ORR resistance (*R*_ORR_), the MOR resistance (*R*_MOR_), and the mass-transport resistance within DMFCs (*R*_trans_), respectively. These four resistances of the DMFC with the Pt/C–PAA–Nafion electrode and the Pt/C–PAA–Nafion electrode are displayed in Figure 5a. The corresponding constant phase elements, denoted as *CPE*_ORR_, *CPE*_MOR_, and *CPE*_trans_, are associated with the ORR, MOR, and mass-transport processes, respectively. Specifically, the *R*_ohmic_ is related to the ohmic polarization losses observed in the IV curves of DMFCs. These losses are determined by the resistance of key components in DMFCs as well as the interfacial resistance between different materials. The *R*_ORR_ and *R*_MOR_ correspond to the activation polarization losses in the IV curves of DMFCs. These resistances are influenced by many factors such as electrocatalyst loadings, the reactant concentration, and the operating temperature. Meanwhile, the *R*_trans_ is concerned with the mass-transport polarization losses in the IV curves of DMFCs, reflecting limitations in the methanol and oxygen transport in DMFCs. Hence, it is urgently needed for the detailed analysis of the above resistances in DMFCs, and the related parameters are summarized in Figure 5c. Notably, the significant discrepancy could be found in the *R*_ohmic_, *R*_ORR_, and *R*_MOR_ for DMFCs assembled with different Pt/C electrodes, suggesting that this improvement in the electrochemical properties should be related to these aspects. Hence, the detailed analysis could further focus on the above aspects.

(1) Analysis of the effect of the conductivity on the performance of DMFCs: Based on the results of Figure 5c, the average *R*_ohmic_ from three EIS measurements of DMFC assembled with the Pt/C–PAA–Nafion electrode is 0.079 Ω cm^2^, with a reduction of 46% compared to that of DMFC assembled with the conventional Pt/C electrode. Given the same components in the anode and the electrolyte membrane, the discrepancy in *R*_ohmic_ could be attributed to the excellent conductivity from the entangled porous nanofiber networks. Further, the ohmic resistance (IR) corrected polarization curves are obtained as displayed in Figure 6. The enhancement in the peak power density is summarized in Table 2 before and after the IR correction. Apparently, the peak-power density of DMFCs after the IR correction slightly outperforms that of the uncorrected values. The larger enhancement values (7.74%, 8.64%, 9.67%, and 6.47%) could be found in DMFCs assembled with the conventional Pt/C electrode. This suggests that the ohmic resistance has a more significant impact on the output performance of DMFCs assembled with the conventional Pt/C electrode than that with the Pt/C–PAA–Nafion electrode. Hence, we could reconsider that the ohmic resistance is one but not the main aspect bringing out the discrepancy in the output performance of DMFCs assembled with the Pt/C–PAA–Nafion electrode.

Furthermore, EIS measurements are performed on both the Pt/C–PAA–Nafion electrode and the conventional Pt/C electrode to evaluate the impact of conductivity on the performance of DMFCs. Although a layer-resolved conductivity analysis using blocked-electrode EIS is more suitable, the effective electrode conductivity and effective proton conductivity are obtained according to the method described in our previous work, which could also be used to analyze the influence of conductivity on the performance of DMFCs [31]. As illustrated in Figure 7a, the average conductivity from three measurements of all electrodes increases with rising temperature. At 60 °C, the Pt/C–PAA–Nafion electrode exhibits a smaller conductivity than the conventional Pt/C electrode. However, at higher temperatures of 70 °C and 80 °C, the Pt/C–PAA–Nafion electrode demonstrates superior conductivity. This suggests more efficient charge transfer within the Pt/C–PAA–Nafion electrode, resulting in lower ohmic resistance in the corresponding DMFC compared to that using the conventional Pt/C electrode. Moreover, the average proton conductivity from three measurements of the Pt/C–PAA–Nafion electrode follows a similar trend with increasing temperature and reaches comparable values to that of the conventional Pt/C electrode, with the highest proton conductivity observed at 80 °C. These findings indicate that the differences in overall conductivity between the two electrodes are primarily due to variations in electron-transfer behavior, thereby influencing the ohmic resistance of DMFCs. Hence, the enhanced electronic conductivity facilitated by the entangled porous-nanofiber networks is identified as the key factor accounting for the reduced ohmic resistance in DMFCs assembled with the Pt/C–PAA–Nafion electrode.

(2) Exploration of the influence of the ORR on the performance of DMFCs: From the results of Figure 5c, the DMFC assembled with the Pt/C–PAA–Nafion electrode demonstrates a smaller average ORR resistance (0.049 Ω cm^2^) from three measurements than the DMFC assembled with the conventional Pt/C electrode (0.088 Ω cm^2^). This indicates that the Pt/C–PAA–Nafion electrode exhibits excellent ORR activity even if the Pt loading (0.068 mg cm^−2^) is less than that of the conventional Pt/C electrode (0.078 mg cm^−2^). Given the effect of triple-phase boundary regions on the electrochemical reactions, the high ORR activity could be related to the macro/mesoscale hierarchical structures within the Pt/C–PAA–Nafion electrode. To explore the effect of the mesoscale hierarchical structures, CV measurements are conducted on DFMCs, as shown in Figure 8. From the CV curves in Figure 8a,b, all CV profiles demonstrate comparable hydrogen desorption/absorption peaks under 0~0.4 V vs. DHE, and the current density of these peaks decreases with the increasing testing temperature. This suggests that similar electrochemical reactions occur in the Pt/C–PAA–Nafion electrode and the conventional Pt/C electrode. Further, the average ECSA could be calculated based on the hydrogen-desorption peak, and the results are displayed in Figure 8c. Notably, the Pt/C–PAA–Nafion electrode exhibits a larger ECSA than that of the conventional Pt/C electrode under all testing temperatures, with an enhancement of 237%, 218%, 244%, and 233%, respectively. More electrochemical regions are provided in the Pt/C–PAA–Nafion electrode, and this improves the utilization of Pt/C electrocatalysts. At the same time, many researchers have evidenced the excellent oxygen-transport properties in the porous-nanofiber-network electrode. Hence, the Nafion/Pt triple-phase reaction regions formed by Pt/C electrocatalyst particles, the Nafion thin film, and the porous structures, together promote the outstanding ORR activity of the Pt/C–PAA–Nafion electrode. Hence, the output performance of DMFCs could be related to the effective triple-phase boundary regions within the Pt/C–PAA–Nafion electrode.
(3) Investigation of the MOR impact on the performance of DMFCs: The open-circuit voltage (OCV) of DMFCs is derived from the IV curves to evaluate the influence of the methanol diffusive transfer through the electrolyte on the performance of DFMCs. As summarized in Table 3, under the testing temperatures of 60 °C, 70 °C, 80 °C, and 90 °C, the OCV of DMFCs assembled with the Pt/C–PAA–Nafion electrode is 0.75, 0.77, 0.75, and 0.74 V, respectively. In comparison, the OCV of the conventional Pt/C electrode is 0.74, 0.75, 0.74, and 0.74 V under the same conditions. Notably, the Pt/C–PAA–Nafion electrode exhibits a slight increase in the OCV of DMFCs across the temperature range. This improvement suggests a partial suppression of the effect of MOR on the cathode potential of DMFCs.

Furthermore, the methanol-transport behaviors within the Pt/C–PAA–Nafion electrode are explored to elucidate its impact on the MOR activity. Contact-angle measurement is first conducted on both the Pt/C–PAA–Nafion electrode and the conventional Pt/C electrode. As shown in Figure 9a, the contact angles for both electrodes decrease over time, indicating that methanol transport occurs within both electrodes. However, the initial contact angles are comparable, suggesting a similar methanol distribution at the electrode–membrane interface. From 10 to 60 min, the Pt/C–PAA–Nafion electrode maintained a larger contact angle. This observation suggests that methanol transport is more impeded in the Pt/C–PAA–Nafion electrode. Therefore, the authors could conclude that the Pt/C–PAA–Nafion electrode partially restricts the transport of methanol solution from the cathode–membrane interface into the cathode catalyst layer. To further analyze the methanol-transport process, the mass of the methanol solution within each electrode is quantified. Figure 9b shows the mass of the Pt/C–PAA–Nafion electrode before and after the transport processes of the methanol solution, with 38% more methanol solution than that of the conventional Pt/C electrode. This result indicates a greater methanol solution uptake within the Pt/C–PAA–Nafion electrode. This suggests that the unique pore structure of the Pt/C–PAA–Nafion electrode facilitates the enhanced methanol solution transport and retention. Finally, the MOR activity of both electrodes is evaluated via CV and LSV to correlate the observed methanol-transport properties with corresponding electrochemical properties. Based on Figure 5c, the average *R*_MOR_ from three measurements of DMFC assembled with the Pt/C–PAA–Nafion electrode is 0.99 Ω cm^2^, with a slight increase of 6.30%~19.3%, compared to that of DMFC assembled with the conventional Pt/C electrode. Many researchers have found that MOR not only occurs in the anode fed with methanol solution but also happens in the cathode due to methanol crossover. Hence, the discrepancy in *R*_MOR_ should be related to the methanol–oxygen reactions in the cathode and anode of DMFCs. For the anode of these DMFCs, CV characterization is conducted to explore the electrochemical properties, as shown in Figure 10. Apparently, all CV curves for the anode of DMFCs with different cathodes display the same redox peaks and comparable peak current density at 60 °C, 70 °C, 80 °C, and 90 °C, respectively. Further, the same PtRu loadings and methanol concentration are found within the anode of two DMFCs. Hence, we could regard that DMFCs with different Pt/C electrodes exhibit comparable methanol–oxygen reactions in the anode, and then the discrepancy in *R*_MOR_ is consistent with the methanol–oxygen reactions in the cathode of DMFCs.

To further investigate the MOR activity in different cathodes, LSV characterization is carried out on the cathode of DMFCs, and the results are shown in Figure 11. The LSV profiles of one electrode reveal that the MOR current density increases notably with increasing testing temperature, which is attributed to enhanced methanol-crossover from the anode of DMFCs. At a fixed testing temperature, the MOR current density rises sharply above approximately 0.4 V, reaching a maximum at 0.6 V. From these LSV profiles, the MOR current density and overpotential are calculated, which are related to the MOR activity in the cathode. As summarized in Figure 11c, the DMFC assembled with the Pt/C–PAA–Nafion electrode exhibits a lower current density and higher overpotential, suggesting suppressed MOR activity within this electrode. This is despite a larger electrochemical surface area (ECSA) of the Pt/C–PAA–Nafion electrode compared to the conventional Pt/C electrode, which would otherwise suggest more available active sites for MOR. Given that electrochemical reactions are influenced by reactant concentration and mass transport processes, the partial suppression in MOR activity is likely governed by methanol-transport behavior within the Pt/C–PAA–Nafion electrode. Furthermore, the hierarchical architecture of continuous nanofibers and bead-like Pt/C–ionomer fibrous networks may partially obstruct methanol transport to the efficient triple-phase reaction regions. One possibility is that this physical restriction locally suppresses MOR activity by limiting methanol access. Alternatively, given the lack of direct-outlet analysis, the observed suppression in MOR activity may stem from enhanced methanol-permeability within the Pt/C–PAA–Nafion electrode, leading to a lower methanol concentration. Regardless of the dominant mechanism, both phenomena may contribute to a reduction in the impact of the methanol-crossover on the electrochemical properties of the cathode, thereby contributing to improved performance of the DMFC with the Pt/C–PAA–Nafion electrode.

### 4.2. The Electrochemical Reaction Mechanism Within the Pt/C–PAA–Nafion Electrode

Based on the above results and analysis, the electrochemical reaction mechanisms of the Pt/C–PAA–Nafion electrode are summarized. The outstanding output performance of the Pt/C–PAA–Nafion electrode in DMFCs is attributed to three key structural features of the hierarchical macro/mesoscale structures: (1) High electronic conductivity facilitated by the entangled-porous nanofiber networks. This partially reduces the ohmic polarization losses as observed in the IV characteristics of DMFCs; (2) Efficient triple-phase boundary regions are formed by Pt/C electrocatalyst particles and the Nafion thin film. This could lead to a larger ECSA, lower oxygen permeation resistance, and enhanced ORR activity even with low Pt loading. (3) Restricted methanol accessibility to triple-phase boundary regions due to the rough surface of Pt/C–PAA–Nafion nanofibers. This contributes to a reduction in the effect of the methanol-crossover on the electrochemical properties of the cathode, the hybrid potential in the cathode, and the output performance of DMFCs. Hence, the Pt/C–PAA–Nafion electrode demonstrates significantly improved electrochemical properties in the DMFC compared to that of the conventional Pt/C electrode

## 5. Conclusions

In this work, we fabricate a Pt/C–PAA–Nafion electrode with hierarchical macro/mesoscale structures via electrospinning. The electrode comprises both bead-like Pt/C–ionomer hybrid porous-nanofibers and continuous smooth nanofibers. SEM, TEM, and TG analyses indicate that the continuous smooth nanofibers in Pt/C-PAA–Nafion nanofibers, with an average diameter of 900 nm, are composed of PAA materials and Nafion materials. In contrast, the hybrid porous nanofibers incorporate PAA materials, Nafion materials, and Pt/C electrocatalysts, forming Nafion/Pt triple-phase reaction regions conducive to ORR and MOR. XPS and electrochemical analyses further reveal the modifications in the electronic state of Pt/C electrocatalysts. Combined with optimized triple-phase reaction regions, these collectively enhance the ECSA and thus the output performance of DMFCs. Meanwhile, restricted methanol from the porous-nanofiber networks to Nafion/Pt triple-phase reaction regions and uneven methanol distribution along the porous-nanofiber networks result in lower current density and higher overpotential of MOR within the Pt/C–PAA–Nafion electrode. These effects partially mitigate the impact of the methanol-crossover on the electrochemical properties of the cathode. As a result, the DMFC assembled with the Pt/C–PAA–Nafion electrode exhibits a maximum mass power density 55% higher than that of the conventional Pt/C electrode. This study demonstrates the application of the Pt/C–PAA–Nafion electrode with hierarchical macro/mesoscale structures in DMFCs and promotes the development of the cathode of DMFCs with high performance and low cost.

## Figures and Tables

**Table 1:** XPS results for the Pt/C–PAA–Nafion electrode and the conventional Pt/C electrode.

Samples	Bonding Energy of Pt 4f/eV				Bonding Energy of O 1s/eV			Bonding Energy of S 2p/eV	
	Pt (II) 4f_5/2_	Pt 4f_5/2_	Pt (II) 4f_7/2_	Pt 4f_7/2_	–OH	C–O–C	–SO_3_	S 2p_3/2_	S 2p_1/2_
-Pt/C–PAA–Nafion	75.75	74.85	72.45	71.55	534.9	533.35	532.0	169.85	168.65
Pt/C electrode	76.65	75.30	73.30	71.95	535.15	533.30	531.80	169.55	168.35

**Table 2:** Peak-power density for DMFCs assembled with different Pt/C electrodes.

Testing Temperature	Peak-Power Density Before IR Corrected/mW cm^−2^		Peak-Power Density After IR Corrected/mW cm^−2^		Enhancement Values	
	Pt/C–PAA–Nafion	Conventional Pt/C	Pt/C–PAA–Nafion	Conventional Pt/C	Pt/C–PAA–Nafion	Conventional Pt/C
60 °C	52.2	43.9	54.4	47.3	4.21%	7.74%
70 °C	58.3	52.1	61.3	56.6	5.15%	8.64%
80 °C	52.7	45.5	55.4	49.9	5.12%	9.67%
90 °C	45.9	34.0	48.8	36.2	6.32%	6.47%

**Table 3:** The open-circuit voltage of DMFCs under different temperatures.

Samples	The Open-Circuit Voltage of DMFCs Under Different Temperatures/V			
	60 °C	70 °C	80 °C	90 °C
-Pt/C–PAA–Nafion	0.75	0.77	0.75	0.74
Pt/C electrode	0.74	0.75	0.74	0.74

## Data Availability

Data are contained within the article.
